# The Protein Quality Control Machinery Regulates Its Misassembled Proteasome Subunits

**DOI:** 10.1371/journal.pgen.1005178

**Published:** 2015-04-28

**Authors:** Lee Zeev Peters, Ofri Karmon, Galit David-Kadoch, Rotem Hazan, Tzenlin Yu, Michael H. Glickman, Shay Ben-Aroya

**Affiliations:** 1 Faculty of Life Sciences Bar-Ilan University, Ramat-Gan, Israel; 2 Departement of Biology, Technion-Israel Institute of Technology, Haifa, Israel; Stanford University, UNITED STATES

## Abstract

Cellular toxicity introduced by protein misfolding threatens cell fitness and viability. Failure to eliminate these polypeptides is associated with various aggregation diseases. In eukaryotes, the ubiquitin proteasome system (UPS) plays a vital role in protein quality control (PQC), by selectively targeting misfolded proteins for degradation. While the assembly of the proteasome can be naturally impaired by many factors, the regulatory pathways that mediate the sorting and elimination of misassembled proteasomal subunits are poorly understood. Here, we reveal how the dysfunctional proteasome is controlled by the PQC machinery. We found that among the multilayered quality control mechanisms, UPS mediated degradation of its own misassembled subunits is the favored pathway. We also demonstrated that the Hsp42 chaperone mediates an alternative pathway, the accumulation of these subunits in cytoprotective compartments. Thus, we show that proteasome homeostasis is controlled through probing the level of proteasome assembly, and the interplay between UPS mediated degradation or their sorting into distinct cellular compartments.

## Author Summary

The accumulation of misfolded proteins threatens cell fitness and viability and their aggregation is commonly associated with numerous neurodegenerative disorders. Cells therefore rely on a number of protein quality control (PQC) pathways to prevent protein aggregation. In eukaryotes, the ubiquitin proteasome system (UPS), a supramolecular machinery that mediates the proteolysis of damaged or misfolded proteins, plays a vital role in PQC by selectively targeting proteins for degradation. Although the critical role-played by the UPS in PQC, and the severe consequences of impairing this pathway are well established, little was known about the mechanisms that control dysfunctional proteasome subunits. Here, we reveal that the interplay between UPS mediated degradation of its own misassembled subunits, and sorting them into cytoprotective compartments, a process that is mediated by the Hsp42 chaperone, determines how proteasome homeostasis is controlled in yeast cells. We believe that the mechanism of proteasome regulation by the PCQ in yeast may serve as a paradigm for understanding how homeostasis of this essential complex is controlled in major chronic neurodegenerative disorders in higher eukaryotes.

## Introduction

Protein homeostasis encompasses the systems required by the cell for generating and maintaining the correct levels, conformational states, distribution, and degradation of its proteome. Maintaining protein homeostasis is crucial to cells given the toxic potential of misfolded proteins and aggregates. Cells therefore rely on a number of protein quality control (PQC) pathways that survey proteins both during and after synthesis to prevent protein aggregation, promote correct protein folding, and target terminally misfolded proteins to degradation. In eukaryotes, the ubiquitin proteasome system (UPS) plays a vital role in PQC by selectively targeting proteins for degradation [1–4].

The eukaryotic 26S proteasome is a highly conserved 2.5-MD multisubunit protease responsible for degrading a large fraction of intracellular proteins. The 26S proteasome comprises a 20S core particle (CP) and two 19S regulatory particles (RP) that are further divided into lid and base complexes [5]. The degradation of most proteins is mediated by polyubiquitin chains labeling, which leads to their recognition by the 26S proteasome [6]. A diverse array of fundamental biological processes are controlled by the UPS, including cell cycle progression, DNA repair, signal transduction, and PQC in which the cell removes abnormal and toxic proteins generated as a result of environmental damage [7,8]. Under such conditions, chaperones are tasked with accompanying terminally misfolded and aggregated proteins to disposal, or to limit inclusion of these proteins, thereby preventing protein aggregates from causing cell toxicity and from being transferred to the next generation [2]. This chaperone mechanism, alongside the UPS, is termed spatial quality control, and consists of the juxtanuclear quality control compartment (JUNQ) and the insoluble protein deposit (IPOD), which were identified in yeast [9,10]. The JUNQ provides a specialized environment for chaperone-mediated refolding or proteasomal protein degradation. Proteins that are not refolded or degraded in the JUNQ are mobilized to the IPOD.

Alongside its PQC role, the UPS plays an essential role in regulating the degradation and function of nuclear proteins [11–13]. Accordingly, immune-electron microscopy experiments [14] and fluorescent microscopy of GFP-tagged proteasome subunits [15] have established that the 26S proteasome is highly enriched in the nucleus. In exponentially growing yeast cells, 80% of the 26S proteasomes are localized inside the nucleus throughout the cell cycle [16]. Given the importance of the UPS, proteasomal nuclear mislocalization may have severe consequences, for example, a deleterious effect on DNA double strand break repair [13].

Although ubiquitin-mediated proteasomal degradation of many proteins plays a key role in the PQC system, the proteasome itself can become dysfunctional as a result of transcriptional and translational failures, genomic mutations, or diverse stress conditions, leading to misfolded proteins existing in every compartment of the cell. The regulatory pathways, and the identity of the cellular machinery that mediates the sorting sequestration and elimination of dysfunctional proteasomal subunits remain poorly understood.

By using a mutated proteasome lid subunit (termed *rpn5ΔC*), we show that the nuclear mislocalization, and the cytosolic aggregates formed by this mutant represent a misassembled proteasome lid. With this experimental tool in hand, we were able to demonstrate how the dysfunctional proteasome is controlled by the PQC machinery. We found that among the multilayered quality control mechanisms, the UPS-mediated degradation of its own dysfunctional subunits is the favored pathway. However, in the absence of a functional proteasome, peripheral aggregates that represent misassembled proteasome accumulate in the IPOD, a process that is mediated by the Hsp42 chaperone. We further demonstrate that while the proteasome structure can tolerate the structural defects of the *rpn5ΔC* mutant and assemble into a functional proteasome, the PQC machinery takes-over, and the mutated protein is spatially removed by the PQC machinery, leading to proteasome dysfunction. Overall, our results demonstrate that proteasome homeostasis is controlled through cellular probing of the quality of proteasome aggregates, and the interplay between UPS-mediated degradation of dysfunctional subunits and alternatively, their accumulation in cytoprotective compartments.

## Results/Discussion

### The nuclear mislocalization of the C-terminal domain truncated Rpn5 results in proteasomal lid misassembly

**Fig 1:**
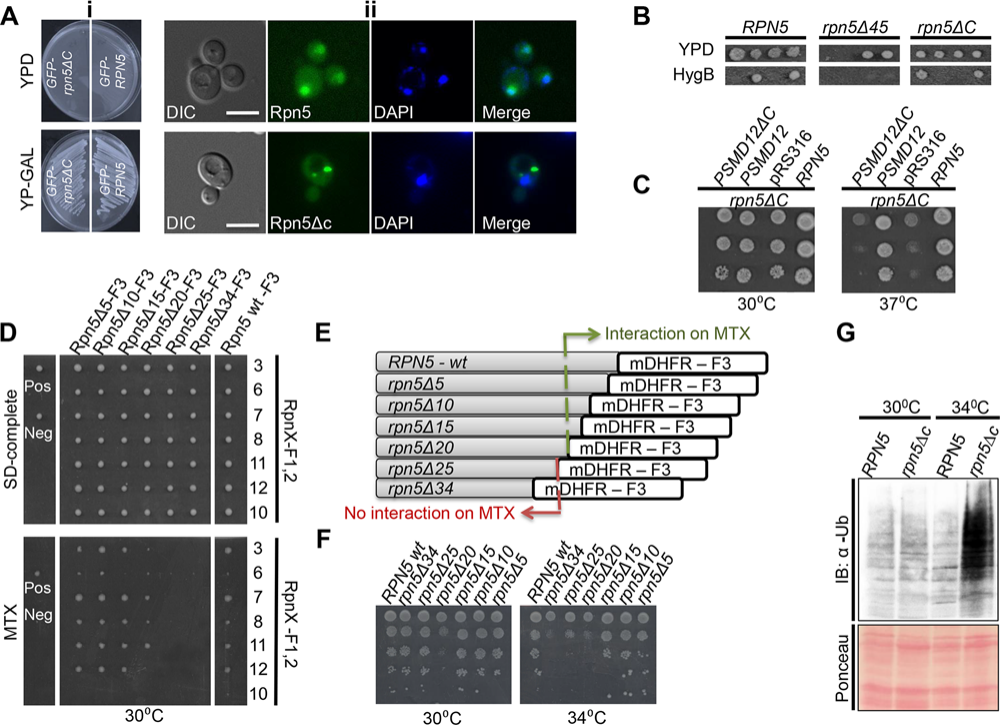
(A) (i) The expression of GFP-*RPN5* through a galactose inducible promoter can fully support cell growth. Cells containing N-terminal fusions of GFP to Rpn5 and Rpn5ΔC, both expressed from a galactose-inducible promoter (*GAL1*-GFP-Rpn5 (SB147), and *GAL1*-GFP-Rpn5ΔC (SB148) respectively), were streaked on glucose (promoter shut-off), and galactose (induced expression) containing medium (YPD and YP-GAL respectively) at the semi-permissive temperature (30°C). (ii) **Truncation at the C-teminus domain (CTD) domain of Rpn5 (Rpn5ΔC) leads to its nuclear mislocalization**. Representative images of the GFP-Rpn5 nuclear localization (*top*), *vs*. the cytoscolic inclusions detected in GFP-Rpn5ΔC (*bottom*). The cells that were described in (i) were grown in 2% galactose-containing medium to logarithmic phase at the semi-permissive temperature (30°C). Cells were visualized by differential interference contrast (DIC) and GFP fluorescence. DAPI was used for nuclear staining. Unless otherwise stated, for all the fluorescent microscopy experiments, pictures represent high resolution (63x) images of a single plane chosen from z series images extending above and below the entire cell. The images shown were selected from at least 200 cells that were visualized in each experiment. Bars 5 μm. (B) **The truncation of 45 amino acids at the C-terminus of Rpn5 (*rpn5*Δ*45*) results in cell death**. To examine how different deletions at the CTD of Rpn5 affect cell growth, we created and sporulated heterozygous diploid strains for either wt, *RPN5* (YSB243X4741), *rpn5*ΔC (34 amino acid truncation) (YSB688X4741), and *rpn5*Δ*45* (45 aa truncation at its CTD) (YSB655). Tetrad dissection and incubation at 30°C showed that the presence of *rpn5*Δ45 in haploids caused cell death, as indicated by failure of spores harboring the hygromycinB (HygB) marker to germinate, when compared to the *RPN5* control, and *rpn5*ΔC. HygB was used as a marker for wt and the truncated forms of *RPN5*. (C) **The truncated human ortholog of *RPN5* (*PSMD12*) fails to complement the temperature sensitivity of *RPN5*Δ*C***. 10-fold serial dilutions of the indicated strains were spotted on SD-uracil medium. Cells were incubated at the semi-permissive (30°C) and non-permissive (37°C) temperatures. All the strains harbor the temperature sensitive allele of *RPN5* (*rpn5*Δ*C)* in the presence of a plasmid expressing either a wt copy of *RPN5* (positive control, LSB2), an empty pRS316 plasmid (negative control, LSB3), a full copy of *PSMD12* (LSB1) or *PSMD12* truncated at its C-terminus (*PSMD12*Δ*C*, YSB106). (D, E) **Truncation of a fragment larger than 20aa from the CTD of Rpn5 impairs its interaction with other proteasomal lid subunits**. (D) **Protein complementation assay [20,43]**. The indicated strains are diploids heterozygote for various truncations (indicated at the x-axis; YSB756, YSB758, YSB861, YSB863, YSB741, YSB688, YSB243) at the C-terminus of *RPN5* fused to mDHFR-F3 (Rpn5ΔX-F3), and one of the proteasomal lid subunits (indicated at the y-axis; YSB671, YSB244, YSB677, YSB245, YSB672, YSB689, YSB670) fused to mDHFR-F1,2 (RpnX-F1, 2). Single colonies were spotted on a rich medium (SD-complete) and allowed to grow for 2 days at 30°C. These colonies were then spotted back to rich medium, and methotrexate (MTX) containing media (30°C). Growth on MTX indicates a positive physical interaction. *CDC19-F1*,*2/CLN3-F3* (YSB59) and *CDC19-F1*,*2/MCK1-F3* (YSB60) diploids were used as negative (Neg) and positive (Pos) controls, respectively. Since it was previously shown that the proteasome lid subunit Rpn10 doesn’t interact with Rpn5, the fusion of Rpn10 to F1,2 (Rpn10-F1,2) was used as an additional negative control. (E) Schematic representation of the different constructs in which one half of the mDHFR fragment (mDHFR-F3) was fused by homologous recombination to the C-terminus of the wt Rpn5 (*RPN5*-wt-F3), and by truncating various aa lengths at its CTD (Rpn5Δ5-F3 to Rpn5Δ34-F3 (termed Rpn5ΔC-F3). The DHFR-F3 fragment is linked to HygB. (F) **Correlation between the extent of truncation at the CTD of *RPN5* and cell viability**. We used a similar experimental setup as in C, this time using the same strains indicated along the x-axis of D, grown at semi-permissive (30°C), and restrictive temperature (34°C). (G) **Lid misassembly in *rpn5ΔC* cells is associated with proteasome dysfunction**. GFP-*RPN5* (*RPN5*) (SB147), and GFP-*rpn5ΔC* (*rpn5ΔC*) (SB148) cells were grown at the semi-permissive (30°C), and samples were transferred to the restrictive temperature (34°C) for 3 hrs. Total protein extracts from both temperatures were subjected to immunoblotting (IB) with anti ubiquitin antibody (α-Ub). Ponceau staining of the blotted protein extracts is shown for loading control.

Recently, we screened a collection of temperature sensitive (Ts) mutants in the yeast *Saccharomyces cerevisiae* for those that show a chromosomal instability (CIN) phenotype [17]. This screen identified proteasome subunit genes, such as the Ts allele of the regulatory particle (RP) subunit *RPN5*, and the core particle (CP) subunit *PUP2*. The Ts allele of *RPN5* was generated by random mutagenesis [17]. Sequence analysis of this mutant revealed that a single base pair insertion resulted in a premature stop codon, leading to a 34 amino acid (aa) truncation at the C-terminal domain (CTD) of Rpn5 (termed *rpn5*Δ*C*). To examine the subcellular localization of Rpn5ΔC, GFP was fused at its N-terminus. An identical N-terminal GFP fusion was constructed for the wt *RPN5* gene, which was used as a control (both fusion proteins were expressed from a galactose-inducible promoter (*GAL1*)). *RPN5* is an essential gene, and therefore, growth on a glucose-containing medium (which shuts-off the expression of both *GAL1*-GFP-*RPN5* (GFP-*RPN5*), and *GAL1*-GFP-*rpn5*Δ*C* (GFP-*rpn5*Δ*C*)) resulted in cell death (Fig 1Ai-*top*). In contrast, when the expression of the wt and the truncated gene were induced by growing the cells on 2% galactose, the growth was fully restored at the semi-permissive temperature (30°C) (Fig 1Ai-*bottom*). Furthermore, as shown in Fig 1Aii-*top*, similarly to the wt proteasome, the control GFP-Rpn5 protein localized predominantly to the nucleus in logarithmically growing cells. These results indicate that GAL1-GFP-*RPN5* fully supports the proteasome function. In contrast, while GFP-Rpn5ΔC cells could still grow at the semi-permissive temperature (30°C), the GFP-Rpn5ΔC protein were detected in cytosolic inclusions in 81% of the cells (n>200) (Fig 1Aii, *bottom*, other localization patterns are shown in S2A Fig).

Next, we wanted to determine whether the mislocalization caused by *rpn5*Δ*C* could be attributed to a failure in proteasome assembly. Previous studies have suggested a model whereby the two parts of the 26S proteasome, namely the CP and RP, are formed and imported to the nucleus independently of each other [18,19]. Our results are in agreement of this model and show that *rpn5*Δ*C* leads to the specific mislocalization of another proteasomal lid subunit (Rpn11) (S1Ai and S1Aii Fig), while the core subunits are retained in the nucleus (S1Aiii and S1Aiv Fig). Similar results were obtained in a reciprocal experiment in which the Ts mutant of the CP *pup2* affects the nuclear enrichment of Pre6-GFP (another CP), while the RP Rpn11-GFP is unaffected (S1B Fig). To further demonstrate the importance of an intact CTD for proteasome integrity, we generated diploid cells in which the original truncation of 34 aa from the CTD was extended to 45 aa (*rpn5*Δ*45*). In this case, no viable haploid *rpn5*Δ*45* spores could be obtained following tetrad dissection (Fig 1B). Moreover, a cross-species complementation experiment revealed that the expression in yeast of a full-length human homolog of *RPN5*, *PSMD12*, but not *psmd12*Δ*C*, was able to rescue the temperature sensitivity of the *rpn5*ΔC strain (Fig 1C).

Next, we wished to test the interaction of truncated *RPN5* with other proteasomal lid subunits. To this end, we used the protein fragment complementation assay (PCA) [20] to examine the interaction between Rpn5ΔC and several other proteasomal lid subunits (Rpn3, Rpn6, Rpn7, Rpn8, Rpn11, and Rpn12) that were previously shown to interact with Rpn5 [7,21,22]. In this assay (for details see S1C Fig) the interaction between two proteins of interest can be detected by cell growth on media in the presence of the dihydrofolate reductase (DHFR) enzyme inhibitor, methotrexate (MTX). The results show that Rpn5ΔC fails to exhibit the expected interactions when compared to the wt Rpn5 control at the semi-pemissive temperature (Fig 1D).

This result was supported biochemically by an immunoprecipitation experiment showing that when Rpn8 fused to a FLAG-Tag (Rpn8-Flag) is pulled-down, it shows a decreased interaction with Rpn5 containing a 34 aa deletion in its CTD (Rpn5ΔC-F[3]) (S1D Fig).

By generating a series of strains with defined deletions at the C-terminus of Rpn5 (Fig 1D and 1E), we next showed that the interaction with other proteasomal lid subunits is impaired only when the deletion at the CTD domain is greater than 20aa. The physiological importance of these interactions is highlighted by the clear correlation between the extent of truncation and cell viability at the restrictive temperature (34°C) (Fig 1F).

Moreover, we analyzed total protein by immunoblotting with anti Ub Abs. The results (Fig 1G) show a clear increase in protein ubiquitination in GFP-Rpn5ΔC cells, when compared to the GFP-Rpn5 control, at the restrictive temperature of 34°C. We therefore concluded that the presence of the truncated form of *RPN5* is associated with proteasome dysfunction.

Our results are in agreement with a previous study showing that in *rpn5-1* cells, containing a different CTD-truncated Ts mutant of *RPN5*, lid subcomplexes are not assembled, even to a partial extent, at the restrictive temperature [18]. This study also specifically examined the effect of the *rpn5-1* mutation on the UPS, by evaluating the stability of three model substrates of the ubiquitin–proteasome pathway. The results demonstrated that compared with the wild-type cells, *rpn5-1* cells maintained the normally short-lived substrates at a higher level at the semi-permissive temperature, indicating that the *rpn5-1* mutation caused a defect in the UPS. [18]. Additional studies mapped the interaction between the subunits of the RP by electron microscopy, tandem mass-spectrometry, affinity purification analysis, and other methods [7,21,23]. These approaches demonstrate that Rpn5, Rpn8, Rpn9, and Rpn11 form a stable soluble subcomplex, and the authors have proposed a subunit interaction map, supporting the notion that Rpn5 is a core component in the lid formation. Furthermore, it was also shown that in yeast, Rpn5 is independently incorporated through its CTD into the proteasomal lid [24].

**Fig 2:**
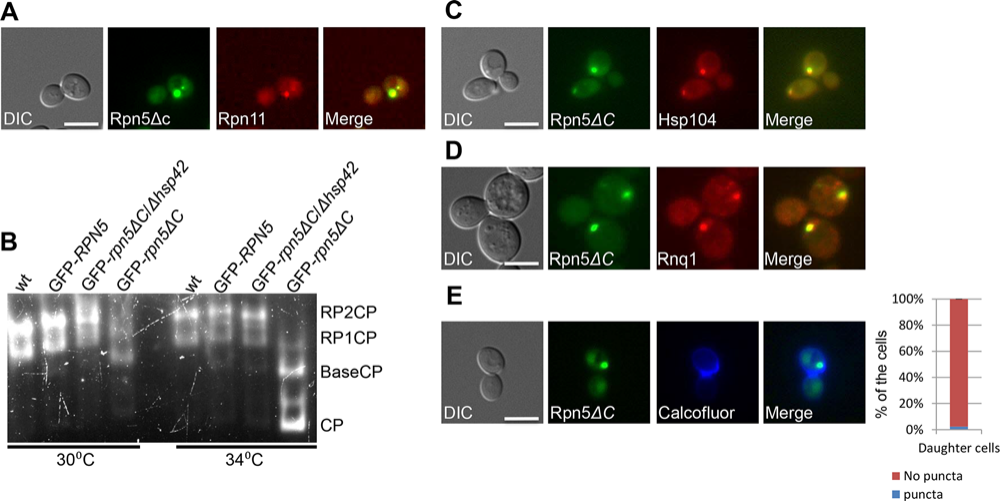
(A) The RP subunit Rpn11-RFP co-localizes with GFP-Rpn5ΔC. Logarithmically growing cells containing Rpn11 fused to RFP (Rpn11-RFP) and GFP-Rpn5ΔC (YSB1090) where grown in galactose containing medium at 30°C and shifted to 34°C for 3 hrs. Cells were visualized by DIC, GFP, and mCherry. (B) **Misassembled lid in *rpn5*Δ*C* is not associated with the proteasome**. Rapidly lysed whole cell extracts from wt (endogenous levels of *RPN5*) (YSB219), and cells over expressing GFP-*RPN5*, or GFP-*rpn5*Δ*C* (SB147, SB148) thorough a galactose inducible promoter (GFP*-RPN5*, and GFP-*rpn5*Δ*C* respectively) were resolved by nondenaturing PAGE, and proteasome visualized by in-gel peptidase activity. *wt* proteasomes are found as a mixture of RP2CP and RPCP. Proteasomes in *rpn5*Δ*C* mutants migrate faster, pointing to a structural defect. Cells were grown at the semi-permissive (30°C), and samples were then transferred to the restrictive temperature (34°C) for 2 hrs. The GFP-*rpn5*Δ*C/*Δ*hsp42* samples are discussed below. (C,D) **Rpn5ΔC cytosolic aggregates co-localize with the IPOD**. Logarithmically growing cells co-expressing GFP*-*Rpn5ΔC, together with the IPOD markers Hsp104-TFP (YSB747) (C) and Rnq1-mCherry (YSB1004) (D), were grown in rich galactose containing medium at 30°C. Hsp104-TFP, and Rnq1-mCherry co-localized with GFP-Rpn5ΔC throughout the experiment. (E) **GFP-Rpn5ΔC large cytosolic aggregates remain sequestered to the mother cells**. Samples in logarithmic growth at the semi-permissive temperature (30°C) were stained by calcofluor. The localization of GFP-Rpn5ΔC (SB148) signal was scored as the percentage of daughter cells issued from IPOD containing mother cells with (puncta), or without (no puncta) cytosolic puncta. A minimum of 100 cells was counted (n >100); error bars show the standard deviation between two independent experiments.

These results, together with our observation that the RP subunit Rpn11-RFP co-localizes with 98% of the cells containing a large GFP-Rpn5ΔC cytosolic aggregate (n>100) (Fig 2A), suggest that these aggregates represent misassembled proteasome lid intermediates.

### Inhibition of 26S proteasome assembly in *rpn5ΔC* mutants

Next, we tested the degree of lid assembly into 26S proteasome holocomplexes in GFP-Rpn5, or GFP-Rpn5ΔC at the semi-permissive and restrictive temperatures using the in-gel peptidase activity assay. In this assay proteasomes are resolved by non-denaturing PAGE according to their charge/mass ratio directly from whole cell extract, and visualized based on inherent peptidase activity as described in [25]. The results (Fig 2B) clearly show that similarly to cells expressing the endogenous levels of Rpn5 in the wt, the over-expression of GFP-Rpn5 by a galactose inducible promoter, had no effect on proteasome integrity, as in both cases similar amounts of proteasomes were found as a mixture of RP2CP, and RP1CP. In contrast, the over production of GFP-Rpn5ΔC resulted in structural defects, as evident by higher levels of RP1CP at the semi permissive temperature (30°C), and free BaseCP and CP mostly at the restrictive temperature (34°C). Similar defects were previously reported by Yu et al, when using *rpn5ΔC* mutant, expressed through its endogenous promoter [24]. Taken together, these results clearly show that proteasome assembly is inhibited in *rpn5ΔC* cells, and that the misassembled lid in *rpn5ΔC* is not associated with active proteasome.

### Misassembled proteasome subunits co-localize with the insoluble protein deposit (IPOD) compartment

Aggregation prone proteins are partitioned between the JUNQ and the IPOD [9,10,26]. Proteins that are ubiquitinated by the PQC machinery are delivered to the JUNQ where they are processed for degradation by the proteasome [9]. We therefore hypothesized that the impairment of the PQC degradation pathway by the *rpn5ΔC* mutant, should lead to the accumulation of misassembled proteasomal lid subunits in the IPOD. To test this idea, we followed the localization of Hsp104, a commonly used IPOD marker [9,10,27], fused to TFP (Hsp104-TFP), in a GFP-Rpn5ΔC strain grown in rich galactose containing medium. Our results show that in all cases where GFP-Rpn5ΔC cytosolic inclusions could be detected (81% of the cells, n>200, S2A Fig), the largest inclusion always co-localized with Hsp104-TFP (n>200) (Fig 2C). Similar results were obtained with the glutamine-rich prion protein Rnq1 fused to mCherry (Rnq1-mCherry), another well-established IPOD marker (Fig 2D) [9,10,27]. Taken together, these findings suggest that peripheral foci containing misassembled Rpn5ΔC are directed to the IPOD when a functional proteasome is scarce, such as in *rpn5ΔC* cells.

It should be noted that since the *RPN5* proteasome subunit is essential for cell viability [17,28], our experiments were performed mainly at the semi-permissive temperature (30°C), to enable its partial (hypomorphic) function. At this temperature, some proteasomes apparently function sufficiently to support cell growth. Indeed, in 19% of the cells (n>200), the GFP-Rpn5ΔC signal was limited to the nucleus, the expected localization in proliferating cells (S2A-*top* Fig). Since the sequestration of aggregates to the IPOD was shown to preclude their delivery by the parental cells to subsequent generations [10], the nuclear GFP-Rpn5ΔC signal, may also represent the functional proteasomes in recently separated daughter cells, as demonstrated in S1 Movie. This idea is also supported by calcofluor staining showing that the GFP-Rpn5ΔC signal was not detected as cytosolic inclusions in 98% of the daughter cells issued from IPOD containing mother cells (Fig 2E).

In addition to the nuclear localization, 81% of cells showed cytosolic inclusions of the GFP-Rpn5**Δ**C signal with the following patterns (representative images are shown in S2A Fig, n>200): 25% exhibited a single large cytosolic aggregate representing the IPOD, and 39% contained a second smaller juxtanuclear protesomal signal, likely representing the JUNQ [9]. Finally, it was recently demonstrated that under acute stress (such as in the case of partially assembled proteasome), misfolded proteins are initially collected and processed in the form of multiple puncta named Q-bodies, which are rapidly reversible structures, that can be dynamically directed to folding and refolding by the chaperone machinery, or to degradation by the proteasome or autophagy system [10,26]. Indeed in 17% of the cells, we detected many cytosolic aggregated bodies, which probably represent these structures.

### The ubiquitin proteasome system mediates the degradation of misassembled proteasomal subunits

**Fig 3:**
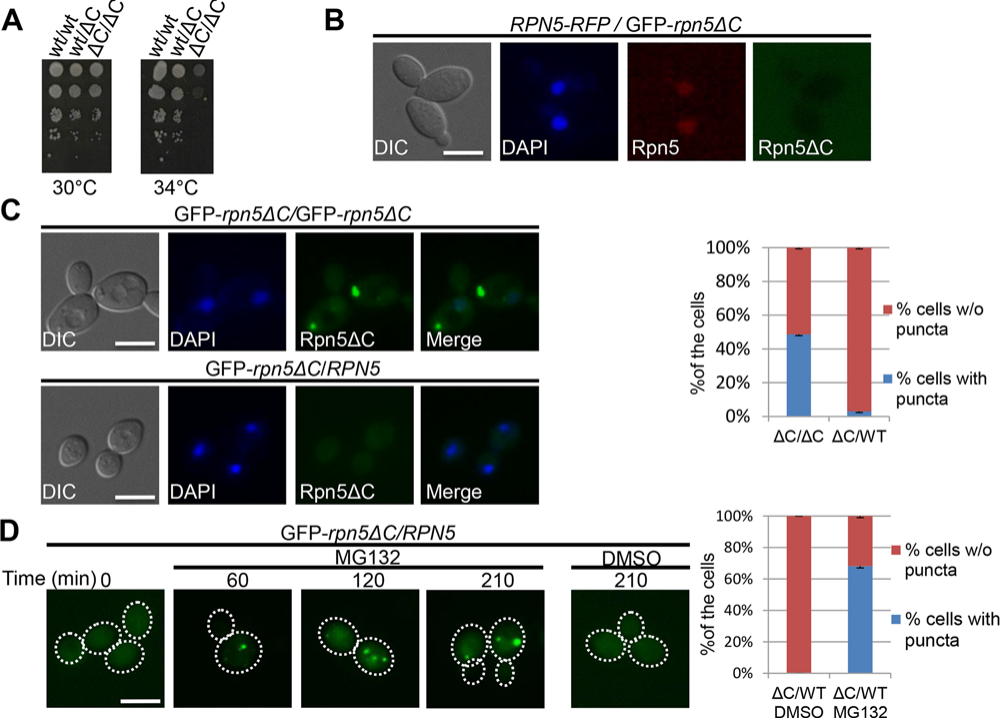
(A) Complementation of *rpn5ΔC* by the wt copy of *RPN5*. 10-fold serial dilutions of diploid strains that are homozygote (ΔC/ΔC, YSB906), or heterozygote (wt/ΔC, YSB908) for the temperature sensitive allele of *RPN5* (*rpn5*Δ*C*). A wt diploid (wt/wt, BY4743) was used as a positive control. Cells were spotted on rich medium, and incubated at the semi-permissive (30°C), and restrictive (34°C) temperatures. (B) **The wt copy of *RPN5* in the GFP-*RPN5ΔC/RPN5*-RFP heterozygous diploid localizes to the nucleus**. Logarithmically growing GFP-Rpn5ΔC/Rpn5-RFP heterozygous diploid cells (YSB1056) were grown in galactose containing medium at 30°C. Cells were visualized by DIC, GFP, DAPI and mCherry. (C) **Complementation of the temperature sensitive phenotype of *rpn5ΔC* by the wt copy of *RPN5* is associated with the elimination of Rpn5ΔC cytosolic aggregates**. Similar to B, but using GFP-*rpn5ΔC*/*RPN5* heterozygotes diploids (YSB908), and GFP*ΔC*/GFP-*rpn5ΔC* homozygous diploids (YSB906) as a control. The graph shows quantitation of the percentage of cells with (blue), or without (w/o) (red) GFP-Rpn5 cytosolic puncta. (D) **Degradation of GFP-Rpn5ΔC cytosolic aggregates is mediated by the proteasome**. The presence of GFP-Rpn5ΔC signal was visualized in GFP-*rpn5ΔC*/*RPN5* heterozygotes diploids (YSB908), in logarithmically growing cells (t-0), and at the indicated time points at 30°C, after the addition of the proteasome inhibitor, MG132, or DMSO (control). The presence or absence of cytosolic puncta was quantitated at the 210 min time point.

One of the challenges faced by the cell in maintaining protein homeostasis is the presence of misfolded proteins. The UPS, in particular, plays a critical role in PQC by selectively targeting proteins for degradation. To test whether the UPS can also regulate the degradation of its own misassembled subunits, *we introduced a functional allele of* RPN5 by mating the haploid GFP-*rpn5ΔC* strain to another haploid containing a wt copy of *RPN5*. The complementation of GFP-*rpn5ΔC* by *RPN5* was indicated by the restoration of growth at the restrictive temperature, and the nuclear localization of the Rpn5 wt protein (Rpn5-RFP) (Fig *3A and 3B). Similarly Rpn11-GFP also localized to the nucleus in* rpn5ΔC/RPN5 *cells (S2B Fig when compared to* rpn5ΔC *haploids (Fig 2A). Interestingly, in the presence of a functional copy of* RPN5, the GFP-Rpn5ΔC puncta was no longer detected in 97% of the cells (n>100) (Fig 3C). Furthermore, the addition of the proteasome inhibitor, MG132, stabilized the GFP-Rpn5ΔC signal, and led to its reproducible accumulation in the IPOD in 68% of the cells (n>100), compared to the DMSO control (Fig 3D). The accumulation of GFP-Rpn5ΔC in cells treated with MG132 was further confirmed by GAL promoter shutoff experiments followed by Western Blot analysis (S2C Fig).

Previous studies suggested that the ubiquitination level of a protein determines whether it is sequestered into the IPOD or JUNQ compartments [9,29]. Impairing misfolded protein ubiquitination blocked their accumulation in the JUNQ, and instead resulted in excessive accumulation in the IPOD [9]. The UPS-mediated degradation of Rpn5ΔC in heterozygote diploids (containing a wt copy of *RPN5*), suggests that Rpn5ΔC is in a ubiquitinated form, when localized to the IPOD. In order to explain this discrepancy, we propose that in *rpn5ΔC* haploids, the cells are under constant acute stress, due to proteasome impairment. Under such conditions, the degradation mechanism is blocked, and therefore there is no alternative, aside from targeting the misassembled lid to the IPOD. This idea is supported by previous reports that proteasome inhibition leads to the accumulation of substrates that are normally degraded by the UPS (such as Ubc9^ts^) into the JUNQ and IPOD [9,26]. In conjunction with this, other studies have demonstrated that when the degradation capacity of the JUNQ declines, with JUNQ-localized proteasomes becoming inactive, a protective alternative is furnished by the IPOD, and toxic aggregating species are rerouted from the JUNQ to the IPOD [30]. We therefore suggest that in our case, when cells fail to degrade misassembled proteasome lid subunits, they can be targeted to the IPOD when still conjugated to ubiquitin.

Taken together, our results suggest that the preferred PQC pathway of misassembled proteasome subunits is degradation by the UPS. As functional proteasomes are scarce in *rpn5ΔC* cells grown at the restrictive temperature, the UPS pathway is hindered. Thus, misassembled lid subunits are ultimately diverted to the IPOD.

### *HSP42*-dependent sequestration of misassembled proteasomal lid subunits precludes the assembly of functional proteasomes

**Fig 4:**
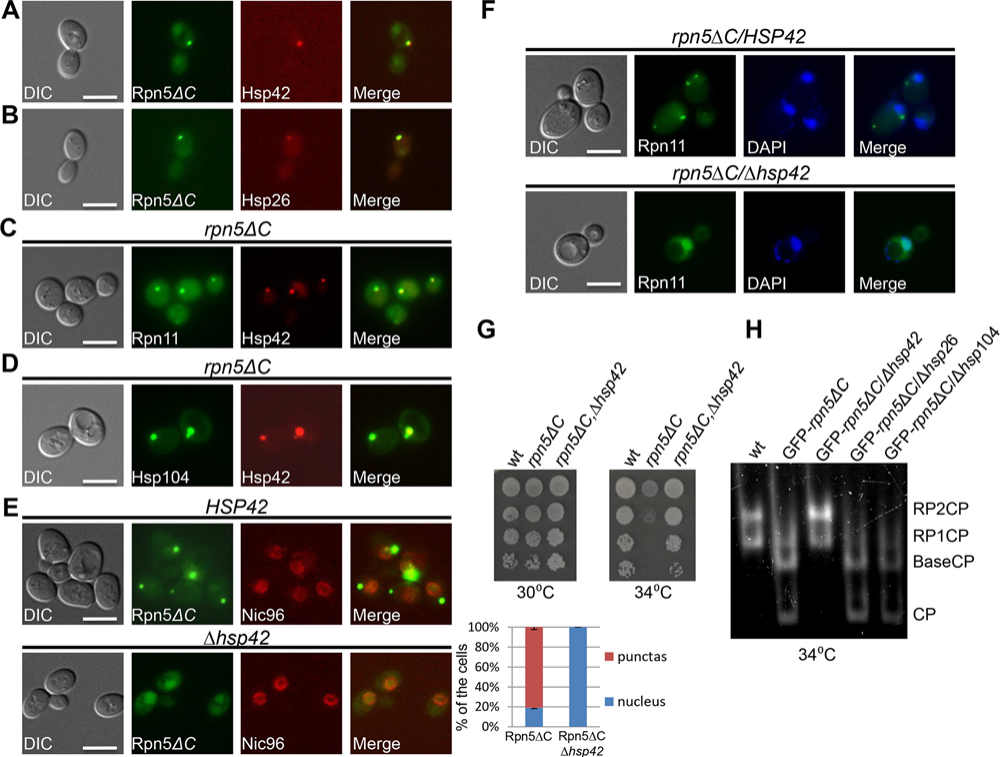
(A-C) Hsp42 and Hsp26 co-localize with misassembled proteasome lid. (A,B) Logarithmically growing cells co-expressing GFP*-*Rpn5ΔC, together with Hsp42-TFP (YSB726) (A), or Hsp26-TFP (YSB903) (B), were grown in galactose containing media at 30°C. (C) Logarithmically growing cells co-expressing rpn5ΔC, *Hsp42-TFP and Rpn11-GFP* were grown in galactose containing media at 30°C and shifted to 34°C for 3 hrs (YSB577). (D) **Co-localization of Hsp42 with the IPOD marker Hsp104 in an *rpn5Δc* background**. Similar to C, but using *rpn5ΔC* cells co-expressing Hsp42-TFP and Hsp104-GFP (YSB792). (E-F) ***HSP42* is essential for the formation of the cytosolic aggregates by misassembled proteasome lid**. (E) Similar to A, but in *HSP42* wt control (YSB1044) (*top*), and *Δhsp42* cells (YSB1045) (*bottom*). The localization of GFP-Rpn5ΔC signal was scored as the percentage of cells showing nuclear localization, or cytosolic puncta (blue and red bars respectively). An mCherry fusion with NIC96, a component of the nuclear pore complex, was used as a nuclear marker. Unless otherwise stated, for each of the graphs, minimum of 200 cells was counted (n >200); error bars show the standard deviation between two independent experiments. (F) Similar to A,B, but in *Δhsp42* (YSB1002) and *HSP42* (SB163) control cells grown at 34°C. DAPI was used for nuclear staining. (G) **The nuclear relocalization of GFP-Rpn5ΔC in *Δhsp42* cells is associated with growth restoration**. 10-fold serial dilutions of the indicated strains (SB147, SB148, YSB868) were spotted on SC media supplemented with galactose (SC-GAL). Cells were incubated at the semi-permissive (30°C) and restrictive (34°C) temperatures. The wt, and strains harboring *rpn5*Δ*C*, were used as positive and negative controls, respectively. (H) **The nuclear re-localization of GFP-Rpn5ΔC in Δ*hsp42* is associated with proteasome reassembly**. Similar to 2B, but in cells over producing GFP-*Rpn5*Δ*C*, and deleted in the indicated chaperones (YSB219, SB148, YSB868, YSB954, YSB1174 respectively). Cells were grown at the semi-permissive temperature of 30°C, and shifted for 2 hrs to the restrictive temperature (34°C).

Molecular chaperones prevent aggregation and misfolding of proteins, and are thus central to maintaining protein homeostasis. Two chaperones that were previously shown to mediate spatial sequestration of misfolded proteins are the small heat shock proteins Hsp26 and Hsp42. These chaperones efficiently co-aggregate with misfolded proteins, thereby altering the properties of protein aggregates and facilitating disaggregation by other chaperones [31]. This process is a key molecular event that determines whether such a protein is sorted to the JUNQ or to a peripheral site [32,33]. As shown in Fig 4A and 4B, both Hsp26 and Hsp42, fused to TFP, colocalized with GFP-Rpn5ΔC in 100% of the cells containing a large cytosolic aggregate, representing the IPOD *(n>200)*. *Similar results were obtained when we investigated the co-localization of Hsp42-TFP with Rpn11-GFP on a* rpn5ΔC *background expressed from its endogenous promoter (* Fig 4C). Since we have shown that Rpn5ΔC co-localizes with Rpn11 (Fig 2A), and that Hsp42 co-localizes with the IPOD marker Hsp104 on a rpn5Δc *background in 95% of the cells (n>200) (* Fig 4D), we conclude that Hsp42 colocalizes with the misassembled proteasome lid in the IPOD. This co-localization is probably associated with physical interactions between Hsp42 and misassembled proteasome subunits, as indicated by the co-immunoprecipitation between the lid subunit Rpn8, and Hsp42 in *rpn5ΔC* cells (S2D Fig).

*Given the role of* HSP42, HSP26 *and* HSP104 *in controlling the* sequestration of protein aggregates into deposition sites [32–34], we hypothesized that the sequestration of GFP-Rpn5ΔC to the IPOD may also depend on these chaperones. To investigate this possibility, we examined the localization of GFP-Rpn5ΔC in *Δhsp42, Δhsp26* and *Δhsp104* cells. In contrast to *Δhsp26*, and *Δhsp104* which only had a minor effect on GFP-Rpn5ΔC cytosolic peripheral focus (S2E and S2F Fig), in cells lacking HSP42, the GFP-Rpn5ΔC signal was no longer observed in the cytosolic periphery. Instead, it showed the nuclear enrichment expected of the wt proteasome (Fig 4E-*bottom*). Similar results were obtained with Rpn11-GFP in a strain mutated in both *HSP42* and *RPN5* (*Δhsp42*, *rpn5Δc*) (Fig 4F-*bottom*). We therefore concluded that association of misassembled proteasome lid subunits with Hsp42 is required for their accumulation in the IPOD. Strikingly, while the deletion of *HSP26*, and *HSP104* had no effect, the nuclear relocalization of GFP-Rpn5ΔC in *Δhsp42* cells was clearly associated with increased survival at 34°C (Fig 4G), and as revealed by the in gel peptidase activity assay, with the reassembly of functional proteasomes (Fig 4H). Taken together, these results show that *HSP42* plays an essential role in mediating the sequestration of misassembled proteasome lid subunits to the IPOD.

**Fig 5:**
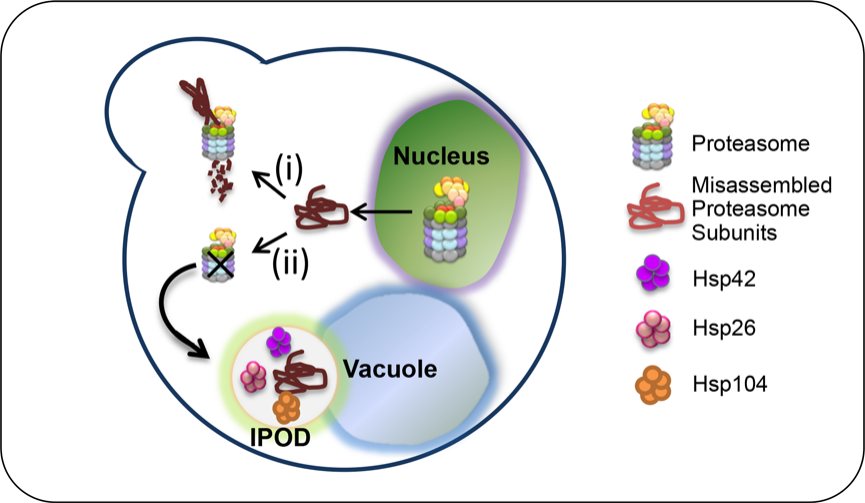
Models: UPS assembly can be naturally impaired by many factors, and thus, there is competition between assembly, degradation and aggregation of proteasome subunits. When the proteasome lids are partially misassembled, as demonstrated in *rpn5ΔC* mutant at the semi-permissive temperature (i), the misassembled subunits are targeted to UPS-mediated degradation by the assembled 26S proteasomes which are still available. This degradation can take place in the nucleus, as recently shown for misfolded proteins [44]. The employment of *rpn5*Δ*C* at the restrictive temperature (ii), shifts the balance to slower assembly, which has a dual effect: It increases the amount of the unassembled subunit, and at the same time decreases its degradation, because there is less proteasomes available. Hence, missassemled proteasome subunits are aggregated in the IPOD, a process that depends mostly on *HSP42*.

Our data is in agreement with previous studies that mapped the interactions between subunits of the proteasome regulatory particles, and led to the notion that Rpn5 is a core component in lid formation [7,21,23], and that in yeast, Rpn5 is independently incorporated through its CTD into the proteasomal lid [24]. Our model (Fig 5) suggests that there is competition between assembly, degradation and aggregation of proteasome subunits. The employment of *rpn5*Δ*C* shifts the balance, since this mutation partially impairs proteasome lid assembly, which in contrast to wt *RPN5*, triggers the activation of the PQC machinery. At the restrictive temperature, the lid is mostly misassembled, and the degradation pathway is blocked, which results in the independent recruitment of Rpn5ΔC, and other lid subunits to the IPOD in an *HSP42* dependent manner. This slower assembly has a dual effect: It increases the amount of the unassembled subunits, and at the same time decreases its degradation, because there is less proteasomes available. Hence, the misassembled subunits aggregate in the IPOD.

The fact that the deletion of *HSP42* restores cell growth at 34°C, suggests that at this temperature, cells can still tolerate the CTD truncation in Rpn5, and assemble partially functional proteasomes (Fig 4G and 4H). A previous study supports this idea by proteasome fractionation using a glycerol gradient, showing that although the truncation influenced integration of additional subunits, Rpn5ΔC could still integrate into the proteasome at the semi-permissive temperature [8]. However, the cell stress imposed by partial misassembly of the lid in the *rpn5* mutant activates the PQC machinery. This activation causes further damage, since lid subunits are independently removed to the IPOD by Hsp42, which in turn leads to complete lid misassembly, proteasome dysfunction, and cell death at the restrictive temperature (34°C). In agreement with this model, the omission of *HSP42* probably prevents the rapid sequestration of Rpn5ΔC into aggregates, allowing more time for 26S proteasomes to assemble, and to degrade the unassembled lid subunits.

Thus, while it was believed that the Rpn5ΔC mutation causes purely structural defects [18], our study provides a plausible alternative mechanism. We suggest that spatial separation of misassembled proteasome lid subunits mediated by the PQC machinery is the key pathway leading to proteasome dysfunction, rather than the structural defects within the *RPN5* mutant.

Taken together, our results reveal that proteins harboring mutations that activate the PQC can be eliminated from the cells, even when the protein is still functional, and the damage ensuing from diverting essential protein products. In light of this, cells have adopted numerous PQC pathways to aid folding, mediate degradation, or to accumulate such proteins in stress foci [3]. This idea is nicely demonstrated by the ΔF508 mutation within the fibrosis transmembrane conductance regulator (CFTR), highly associated with cystic fibrosis (CF) [35]. Although the mutated protein retains significant chloride-channel function, the protein is rapidly recognized by the PQC machinery and is degraded shortly after synthesis, before it can reach its site of activity at the cell surface [36].

### Concluding remarks

Although the critical role-played by the UPS in PQC, and the severe consequences of impairing this pathway are well established, little was known about the mechanisms that control dysfunctional proteasome subunits. Our results demonstrate for the first time that proteasome homeostasis is controlled through the interplay between UPS mediated degradation of its misassembled subunits, and sorting into the IPOD, a process that is mediated by the Hsp42 chaperone, which determines how proteasome homeostasis is controlled in yeast cells.

The assembly of the proteasome is an intricate process due to the number of subunits that must associate to form an active complex. We used a synthetic mutant that induces proteasome dysfunction. However, the UPS function can be naturally impaired by many factors, including mutations, errors during transcription, RNA processing and translation, trapping of a folding intermediate, incorrect incorporation into multimeric complexes or oxidative damage, all of which are processes that are accelerated during aging [37]. Dysregulation of this pathway results in intracellular deposits of ubiquitin protein conjugates which can be seen in age-related pathologies and in all the major chronic neurodegenerative disorders such as Alzheimer’s, Parkinson’s and Huntington’s diseases as well as amyotrophic lateral sclerosis (ALS) and others [37] [38].

The mechanism of proteasome regulation by the PQC in yeast may serve as a paradigm to understand how homeostasis of this essential complex is controlled in higher eukaryotes. Identifying additional chaperones that work in conjunction with Hsp42, and elucidating the identity of the structurally abnormal features that the PQC machinery recognizes in the misassembled proteasome, will provide further insight into the recognition and targeting mechanisms of dysfunctional proteasomes in cells.

## Materials and Methods

### Yeast strains, plasmids, and growth conditions

Unless otherwise stated, all the strains used in this study are isogenic to BY4741, BY4742, or BY4743 [39]. The relevant genotypes are presented in S1 Table. Deletions, GFP, TFP, and mCherry fusions were generated using one step PCR mediated homologous recombination as previously described [40,41]. For all deletions, the selection markers replaced the coding region of the targeted genes. GFP, TFP and mCherry were fused at the 3’ end of the coding region of the targeted genes, by replacement of their stop codons [40,41]. A *GAL1* promoter was placed at the N-terminal of *RPN5* and *RPN5ΔC* by replacement of their start codon. We have shown that the expression of the fusion protein *GAL1*-GFP-*RPN5* has no effect on proteasome normal phenotype (Fig 1A). Strains containing different mDHFR-F[1,2], and mDHFR-F[3] C-terminal fusion proteins were obtained from the PCA collection (commercially available from Open Biosystems), or in cases in which the strains were absent from the collection, by one step PCR mediated homologous recombination, as described by Tarassov, K, *et al*. [20]. For C-terminal truncations by mDHFR-F3 (Fig 2D and 2E), this fragment was targeted to replace the truncated amino acids at the 3’ end.

#### Plasmids

The plasmids used to express *PSMD12*, the human homolog of *RPN5* were generated using the Gateway Technology (available from Invitrogen). Human cDNA of *PSMD12* (Open Biosystems Cat#BC019062) was used as a template for the PCR reactions. PCR products were first cloned into pDONR221 (Invitrogen cat# 12536–017) using the BP recombination reaction (see Invitrogen manual). The LR recombination reaction was then used for cloning into the pYES-DEST52 destination vector (cat#12286019), for expressing *PSMD12 or PSMD12ΔC* under the control of the *GAL1* promoter in yeast. The p*ESC*::*GAL1-RNQ1-*mCherry, *LEU2* marked plasmid was a kind gift from D. Kaganovich’s laboratory (The Hebrew University, Jerusalem, Israel) [9].

#### Growth conditions

Yeast cells were grown in synthetic complete medium (SC; 0.17% yeast nitrogen base, 0.5% (NH_4_)_2_SO_4_, and amino acids), supplemented with either 2% glucose (SD), or galactose (SC-GAL). Unless otherwise stated, cells were grown at 30°C. As described previously [42,43], for logarithmic culture, cells were grown for 16–18 h and then back diluted 10x with fresh media and allowed to grow for 2 hrs. Proteasomes were inhibited by adding 20μM of the proteasome inhibitor MG132 (prepared from an 800μM MG132 in DMSO stock solution, Sigma), in strains deleted in the *ERG6* gene, to prevent the cells from pumping the drug out of the cell. Standard YEP medium (1% yeast extract, 2% Bacto Peptone) supplemented with, 2% galactose (YP-GAL), or 2% dextrose (YPD) was used for nonselective growth. 2% Bacto Agar was added for solid media.

### Microscopy

Microscopy was performed as previously described [42]. Briefly, cells were observed in a fully automated inverted microscope (Zeiss observer. Z1 Carl Zeiss, Inc.) equipped with an MS-2000 stage (Applied Scientific Instrumentation), a Lambda DG-4 LS 300 W xenon light source (Sutter Instrument), a 63x Oil 1.4 NA Plan-Apochromat objective lens, and a six-position filter cube turret with a GFP filter (excitation, BP470/40; emission, BP525/50; Beamsplitter, FT495), a HcRed filter (excitation, BP592/24; emission, BP675/100; Beamsplitter, FT615), and a DAPI filter (excitation, G365; emission, BP445/50; Beamsplitter, FT395) from Chroma Technology Corp. Images were acquired using a CoolSnap HQ^2^ camera (Roper Scientific). The microscope, camera and shutters (Uniblitz) were controlled by AxioVision Rel. 4.8.2. Images are a single plane of z-stacks performed using a 0.5 μm step.

### PCA assay

The PCA was performed as described previously [20]. Strains were mated on YPD, and diploids were selected on YPD supplemented with clonNAT and hygB. SD supplemented with noble agar (Difco), and methotrexate (MTX; Bioshop Canada) was used for the final selection steps. Drugs were added to the following final concentrations: clonNAT (100 μg/ml, Werner Bioagents); MTX (200 μg/ml (prepared from a 10 mg/ml methotrexate in DMSO stock solution, Bioshop Canada); and HygromycinB (100 μg/ml, Calbiochem).

### Co-immunoprecipitations and western blot analysis

Co-immunoprecipitations and Western blot analysis were carried out as described previously [43]. The antibodies used for the Western blot analysis were anti-DHFR-[F3] (Sigma), anti-FLAG (Sigma), anti-Ubiquitin (Dako Dk-z045801-2), anti-GFP (Roche 11–814460001).

### Native-PAGE

Cultures were grown overnight and washed twice with DDW and once with chilled buffer A (25 mM Tris [pH 7.4], 10 mM MgCl2, 10% glycerol, 1 mM ATP, and 1 mM dithiothreitol [DTT]). Pellet was resuspended in two volumes of buffer A and lysed using glass beads at 4°C. Native lysates were clarified by centrifugation at 16,000×g for 15 min.

### Peptidase activity

Proteasome peptidase activity was studied in native PAGE using the substrate succinyl-LLVY-7-amido-4-methylcoumarinfluorescent peptide (Bachem, Bubendorf, Switzerland) as previously published [24,25].

## Supporting Information

S1 Fig The *rpn5ΔC* mutant leads to the specific misassembly of the proteasomal lid. (A-B) The following images depict how regulatory particle (RP) *rpn5ΔC* or core particle (CP) *pup2-*Ts mutants affect the localization of other proteasome RP, Rpn11-GFP, or CP Pre6-GFP. (A) i, ii**-**Rpn11-GFP is located in the nucleus in wt, but not *rpn5ΔC* (SB158, SB162). Sub-panel iii, iv-Nuclear enrichment of the (CP) Pre6-GFP is not affected by the *rpn5* mutation (SB160, SB163). (B) Similar results in a reciprocal experiment. *pup2*-Ts mutant (CP) (SB223) affects the nuclear enrichment of Pre6-GFP [another (CP)], while (the RP) Rpn11-GFP is unaffected (SB220). (C) Principles of the protein complementation assay (PCA) approach. The strategy used here is based on a mutated version of the murine dihydrofolate reductase enzyme (mDHFR). The mDHFR is split into two complementary fragments (mDHFR-F[1,2], and mDHFR-F[3]) and inserted at the C-terminus of the two genes of interest (X, and Y). The functional copy of the mDHFR confers resistance to the DHFR inhibitor, methotrexate (MTX), which inhibits the native *Saccharomyces cerevisiae* DHFR (yDHFR). Thus, the interaction between the X and Y can be detected as cell growth on media in the presence of MTX. (D) Rpn5ΔC (34 aa truncation) shows decreased interaction with Rpn8-FLAG. Doubly tagged *rpn5ΔC*-F3/*RPN8*-FLAG (YSB1061); *RPN5-*F3/Rpn8-FLAG (YSB675) haploid strains, and the singly tagged *rpn5ΔC*-F3 (YSB688); *RPN5-*F3 (YSB243) control strains, were subjected to immunoprecipitation (IP) with an anti-FLAG antibody. Whole cell protein extracts (WCE), and IP samples, were subjected to immunoblotting (IB) with anti-FLAG and anti-mDHFR-F3 antibodies.
(PPTX)

S2 Fig (A) Representative images showing the different localization patterns of the GFP-Rpn5ΔC signal at the semi-permissive temperature. Logarithmic cells expressing GFP-Rpn5**Δ**C (YSB1044) were grown in galactose containing media at the semi-permissive temperature (30°C). The localization of GFP-Rpn5ΔC was scored as nuclear, IPOD only, IPOD and JUNQ (pointed out by white arrows), or cells containing more than 2 puncta, probably representing the Q-bodies. A minimum of 200 cells was counted (n>200); error bars show the standard deviation between two independent experiments. Bars, 5 μm. (B) The wt copy of *RPN11* in the *rpn5ΔC/RPN5* heterozygous diploid re-localized to the nucleus. Logarithmic *rpn5ΔC/RPN5* cells at the semi-permissive temperature (30°C), containing the RP subunit Rpn11 fused to GFP (Rpn11), and Hsp42 fused to TFP (Hsp42) (YSB577X4742) were grown in rich medium. Cells were visualized by DIC, GFP and mCherry. (C) Western blot detects the levels of GFP-*rpn5ΔC* following a *GAL1* promoter shut-off chase experiment. The expression of *GAL1*-GFP-*rpn5ΔC* (GFP-*rpn5ΔC)* was induced in the indicated strains (YSB906, YSB908), by growing the cells at the semi-permissive temperature (30°C) in 2% galactose (Gal) for 2 hrs (t-0). Cells were released into 2% glucose to shut-off the expression of GFP-*rpn5ΔC*, and samples were collected at timely intervals. Glucose was supplemented with 20mM MG132, or with DMSO (control). Protein extracts were immunoblotted (IB) with α-GFP antibody. Ponceau staining of the blotted protein extracts is shown for loading control. (D) Physical interactions between Hsp42 and the misassembled proteasome subunits Rpn8. wt (YSB219), *HSP42*-GFP/*RPN5* (YSB748), and *HSP42*-GFP/*rpn5ΔC* (YSB1191) cells grown at the semi-permissive temperature (30°C) were subjected to immunoprecipitation (IP) with an anti-GFP antibody. Whole cell protein extracts (WCE), and IP samples, were subjected to immunoblotting (IB) with anti-GFP and anti-Rpn8 antibodies. (E,F) Rpn5ΔC cytosolic aggregates formation was not affected by the deletions of *HSP26*, and *HSP104*. Representative images of logarithmically growing cells expressing GFP*-*Rpn5ΔC in *Δhsp104* (YSB1174), and *Δhsp26* (YSB903) backgrounds, grown in rich galactose containing medium at 30°C.
(PPTX)

S1 Movie The IPOD precludes the delivery of misassembled proteasome by the parental cells to subsequent generations. Yeast cells expressing GFP-Rpn5*Δ*C, (SB148) were grown in SC-GAL. Images were analyzed by time-lapse fluorescent microscopy (Zeiss observer. Z1 Carl Zeiss, Inc.). Frames were taken every 10 min for 2.5 hrs.
(MOV)

S1 Table Yeast strains used in this study. (DOCX)

## References

[1] Su H, Wang X (2010) The ubiquitin-proteasome system in cardiac proteinopathy: a quality control perspective. Cardiovasc Res 85: 253–262. 10.1093/cvr/cvp287 19696071 PMC2797449

[2] Kastle M, Grune T (2012) Interactions of the proteasomal system with chaperones: protein triage and protein quality control. Prog Mol Biol Transl Sci 109: 113–160. 10.1016/B978-0-12-397863-9.00004-3 22727421

[3] Comyn SA, Chan GT, Mayor T (2014) False start: Cotranslational protein ubiquitination and cytosolic protein quality control. J Proteomics 100C: 92–101. 10.1016/j.jprot.2013.08.005 23954725

[4] Amm I, Sommer T, Wolf DH (2014) Protein quality control and elimination of protein waste: the role of the ubiquitin-proteasome system. Biochim Biophys Acta 1843: 182–196. 10.1016/j.bbamcr.2013.06.031 23850760

[5] Hershko A, Ciechanover A (1998) The ubiquitin system. Annu Rev Biochem 67: 425–479. 9759494 10.1146/annurev.biochem.67.1.425

[6] Tanaka K (2009) The proteasome: overview of structure and functions. Proc Jpn Acad Ser B Phys Biol Sci 85: 12–36. 19145068 10.2183/pjab.85.12 PMC3524306

[7] Fukunaga K, Kudo T, Toh-e A, Tanaka K, Saeki Y (2010) Dissection of the assembly pathway of the proteasome lid in Saccharomyces cerevisiae. Biochem Biophys Res Commun 396: 1048–1053. 10.1016/j.bbrc.2010.05.061 20471955

[8] Ciechanover A (2005) Intracellular protein degradation: from a vague idea thru the lysosome and the ubiquitin-proteasome system and onto human diseases and drug targeting. Cell Death Differ 12: 1178–1190. 16094394 10.1038/sj.cdd.4401692

[9] Kaganovich D, Kopito R, Frydman J (2008) Misfolded proteins partition between two distinct quality control compartments. Nature 454: 1088–1095. 10.1038/nature07195 18756251 PMC2746971

[10] Spokoini R, Moldavski O, Nahmias Y, England JL, Schuldiner M, et al (2012) Confinement to organelle-associated inclusion structures mediates asymmetric inheritance of aggregated protein in budding yeast. Cell Rep 2: 738–747. 10.1016/j.celrep.2012.08.024 23022486

[11] Brooks CL, Gu W (2003) Ubiquitination, phosphorylation and acetylation: the molecular basis for p53 regulation. Curr Opin Cell Biol 15: 164–171. 12648672 10.1016/s0955-0674(03)00003-6

[12] Nasmyth K (2002) Segregating sister genomes: the molecular biology of chromosome separation. Science 297: 559–565. 12142526 10.1126/science.1074757

[13] Ben-Aroya S, Agmon N, Yuen K, Kwok T, McManus K, et al (2010) Proteasome nuclear activity affects chromosome stability by controlling the turnover of Mms22, a protein important for DNA repair. PLoS Genet 6: e1000852 10.1371/journal.pgen.1000852 20174551 PMC2824753

[14] Wilkinson CR, Wallace M, Morphew M, Perry P, Allshire R, et al (1998) Localization of the 26S proteasome during mitosis and meiosis in fission yeast. EMBO J 17: 6465–6476. 9822592 10.1093/emboj/17.22.6465 PMC1170994

[15] Enenkel C, Lehmann A, Kloetzel PM (1999) GFP-labelling of 26S proteasomes in living yeast: insight into proteasomal functions at the nuclear envelope/rough ER. Mol Biol Rep 26: 131–135. 10363659 10.1023/a:1006973803960

[16] Russell SJ, Steger KA, Johnston SA (1999) Subcellular localization, stoichiometry, and protein levels of 26 S proteasome subunits in yeast. J Biol Chem 274: 21943–21952. 10419517 10.1074/jbc.274.31.21943

[17] Ben-Aroya S, Coombes C, Kwok T, O'Donnell KA, Boeke JD, et al (2008) Toward a comprehensive temperature-sensitive mutant repository of the essential genes of Saccharomyces cerevisiae. Mol Cell 30: 248–258. 10.1016/j.molcel.2008.02.021 18439903 PMC4130347

[18] Isono E, Nishihara K, Saeki Y, Yashiroda H, Kamata N, et al (2007) The assembly pathway of the 19S regulatory particle of the yeast 26S proteasome. Mol Biol Cell 18: 569–580. 17135287 10.1091/mbc.E06-07-0635 PMC1783769

[19] Li X, Kusmierczyk AR, Wong P, Emili A, Hochstrasser M (2007) beta-Subunit appendages promote 20S proteasome assembly by overcoming an Ump1-dependent checkpoint. EMBO J 26: 2339–2349. 17431397 10.1038/sj.emboj.7601681 PMC1864979

[20] Tarassov K, Messier V, Landry CR, Radinovic S, Serna Molina MM, et al (2008) An in vivo map of the yeast protein interactome. Science 320: 1465–1470. 10.1126/science.1153878 18467557

[21] Sharon M, Taverner T, Ambroggio XI, Deshaies RJ, Robinson CV (2006) Structural organization of the 19S proteasome lid: insights from MS of intact complexes. PLoS Biol 4: e267 16869714 10.1371/journal.pbio.0040267 PMC1523230

[22] Sha Z, Yen HC, Scheel H, Suo J, Hofmann K, et al (2007) Isolation of the Schizosaccharomyces pombe proteasome subunit Rpn7 and a structure-function study of the proteasome-COP9-initiation factor domain. J Biol Chem 282: 32414–32423. 17761670 10.1074/jbc.M706276200 PMC3012426

[23] Fu H, Reis N, Lee Y, Glickman MH, Vierstra RD (2001) Subunit interaction maps for the regulatory particle of the 26S proteasome and the COP9 signalosome. EMBO J 20: 7096–7107. 11742986 10.1093/emboj/20.24.7096 PMC125776

[24] Yu Z, Kleifeld O, Lande-Atir A, Bsoul M, Kleiman M, et al (2011) Dual function of Rpn5 in two PCI complexes, the 26S proteasome and COP9 signalosome. Mol Biol Cell 22: 911–920. 10.1091/mbc.E10-08-0655 21289098 PMC3069016

[25] Leggett DS, Glickman MH, Finley D (2005) Purification of proteasomes, proteasome subcomplexes, and proteasome-associated proteins from budding yeast. Methods Mol Biol 301: 57–70. 15917626 10.1385/1-59259-895-1:057

[26] Escusa-Toret S, Vonk WI, Frydman J (2013) Spatial sequestration of misfolded proteins by a dynamic chaperone pathway enhances cellular fitness during stress. Nat Cell Biol 15: 1231–1243. 10.1038/ncb2838 24036477 PMC4121856

[27] Hill SM, Hao X, Liu B, Nystrom T (2014) Life-span extension by a metacaspase in the yeast Saccharomyces cerevisiae. Science 344: 1389–1392. 10.1126/science.1252634 24855027

[28] Glickman MH, Rubin DM, Fried VA, Finley D (1998) The regulatory particle of the Saccharomyces cerevisiae proteasome. Mol Cell Biol 18: 3149–3162. 9584156 10.1128/mcb.18.6.3149 PMC108897

[29] Amen T, Kaganovich D (2015) Dynamic droplets: the role of cytoplasmic inclusions in stress, function, and disease. Cell Mol Life Sci 72: 401–415. 10.1007/s00018-014-1740-y 25283146 PMC11113435

[30] Weisberg SJ, Lyakhovetsky R, Werdiger AC, Gitler AD, Soen Y, et al (2012) Compartmentalization of superoxide dismutase 1 (SOD1G93A) aggregates determines their toxicity. Proc Natl Acad Sci U S A 109: 15811–15816. 22967507 10.1073/pnas.1205829109 PMC3465386

[31] Haslbeck M, Miess A, Stromer T, Walter S, Buchner J (2005) Disassembling protein aggregates in the yeast cytosol. The cooperation of Hsp26 with Ssa1 and Hsp104. J Biol Chem 280: 23861–23868. 15843375 10.1074/jbc.M502697200

[32] Specht S, Miller SB, Mogk A, Bukau B (2011) Hsp42 is required for sequestration of protein aggregates into deposition sites in Saccharomyces cerevisiae. J Cell Biol 195: 617–629. 10.1083/jcb.201106037 22065637 PMC3257523

[33] Malinovska L, Kroschwald S, Munder MC, Richter D, Alberti S (2012) Molecular chaperones and stress-inducible protein-sorting factors coordinate the spatiotemporal distribution of protein aggregates. Mol Biol Cell 23: 3041–3056. 10.1091/mbc.E12-03-0194 22718905 PMC3418301

[34] Shiber A, Breuer W, Brandeis M, Ravid T (2013) Ubiquitin conjugation triggers misfolded protein sequestration into quality control foci when Hsp70 chaperone levels are limiting. Mol Biol Cell 24: 2076–2087. 10.1091/mbc.E13-01-0010 23637465 PMC3694792

[35] Kerem B, Rommens JM, Buchanan JA, Markiewicz D, Cox TK, et al (1989) Identification of the cystic fibrosis gene: genetic analysis. Science 245: 1073–1080. 2570460 10.1126/science.2570460

[36] Rowe SM, Miller S, Sorscher EJ (2005) Cystic fibrosis. N Engl J Med 352: 1992–2001. 15888700 10.1056/NEJMra043184

[37] Wolfe KJ, Cyr DM (2011) Amyloid in neurodegenerative diseases: friend or foe? Semin Cell Dev Biol 22: 476–481. 10.1016/j.semcdb.2011.03.011 21458579 PMC3182296

[38] Rott R, Szargel R, Haskin J, Bandopadhyay R, Lees AJ, et al (2011) alpha-Synuclein fate is determined by USP9X-regulated monoubiquitination. Proc Natl Acad Sci U S A 108: 18666–18671. 10.1073/pnas.1105725108 22065755 PMC3219120

[39] Brachmann CB, Davies A, Cost GJ, Caputo E, Li J, et al (1998) Designer deletion strains derived from Saccharomyces cerevisiae S288C: a useful set of strains and plasmids for PCR-mediated gene disruption and other applications. Yeast 14: 115–132. 9483801 10.1002/(SICI)1097-0061(19980130)14:2<115::AID-YEA204>3.0.CO;2-2

[40] Goldstein AL, McCusker JH (1999) Three new dominant drug resistance cassettes for gene disruption in Saccharomyces cerevisiae. Yeast 15: 1541–1553. 10514571 10.1002/(SICI)1097-0061(199910)15:14<1541::AID-YEA476>3.0.CO;2-K

[41] Longtine MS, McKenzie A, 3rd, Demarini DJ, NG Shah, A Wach, et al (1998) Additional modules for versatile and economical PCR-based gene deletion and modification in Saccharomyces cerevisiae. Yeast 14: 953–961. 9717241 10.1002/(SICI)1097-0061(199807)14:10<953::AID-YEA293>3.0.CO;2-U

[42] Peters LZ, Hazan R, Breker M, Schuldiner M, Ben-Aroya S (2013) Formation and dissociation of proteasome storage granules are regulated by cytosolic pH. J Cell Biol 201: 663–671. 10.1083/jcb.201211146 23690178 PMC3664706

[43] Lev I, Volpe M, Goor L, Levinton N, Emuna L, et al (2013) Reverse PCA, a systematic approach for identifying genes important for the physical interaction between protein pairs. PLoS Genet 9: e1003838 10.1371/journal.pgen.1003838 24130505 PMC3794912

[44] Park SH, Kukushkin Y, Gupta R, Chen T, Konagai A, et al (2013) PolyQ proteins interfere with nuclear degradation of cytosolic proteins by sequestering the Sis1p chaperone. Cell 154: 134–145. 10.1016/j.cell.2013.06.003 23791384

