# Supplementary material for: The Protein Quality Control Machinery Regulates Its Misassembled Proteasome Subunits
Source: PLoS Genet. 2015 Apr 28;11(4):e1005178. doi: 10.1371/journal.pgen.1005178 (PMC4412499; doi:10.1371/journal.pgen.1005178)
Supplement: S1 Fig — (A-B) The following images depict how regulatory particle (RP) rpn5ΔC or core particle (CP) pup2-Ts mutants affect the localization of other proteasome RP, Rpn11-GFP, or CP Pre6-GFP. (A) i, ii-Rpn11-GFP is located in the nucleus in wt, but not rpn5ΔC (SB158, SB162). Sub-panel iii, iv-Nuclear enrichment of the (CP) Pre6-GFP is not affected by the rpn5 mutation (SB160, SB163). (B) Similar results in a reciprocal experiment. pup2-Ts mutant (CP) (SB223) affects the nuclear enrichment of Pre6-GFP [another (CP)], while (the RP) Rpn11-GFP is unaffected (SB220). (C) Principles of the protein complementation assay (PCA) approach. The strategy used here is based on a mutated version of the murine dihydrofolate reductase enzyme (mDHFR). The mDHFR is split into two complementary fragments (mDHFR-F[1,2], and mDHFR-F[3]) and inserted at the C-terminus of the two genes of interest (X, and Y). The functional copy of the mDHFR confers resistance to the DHFR inhibitor, methotrexate (MTX), which inhibits the native Saccharomyces cerevisiae DHFR (yDHFR). Thus, the interaction between the X and Y can be detected as cell growth on media in the presence of MTX. (D) Rpn5ΔC (34 aa truncation) shows decreased interaction with Rpn8-FLAG. Doubly tagged rpn5ΔC-F3/RPN8-FLAG (YSB1061); RPN5-F3/Rpn8-FLAG (YSB675) haploid strains, and the singly tagged rpn5ΔC-F3 (YSB688); RPN5-F3 (YSB243) control strains, were subjected to immunoprecipitation (IP) with an anti-FLAG antibody. Whole cell protein extracts (WCE), and IP samples, were subjected to immunoblotting (IB) with anti-FLAG and anti-mDHFR-F3 antibodies. (PPTX) [file pgen.1005178.s001.pptx]

## Slide 1
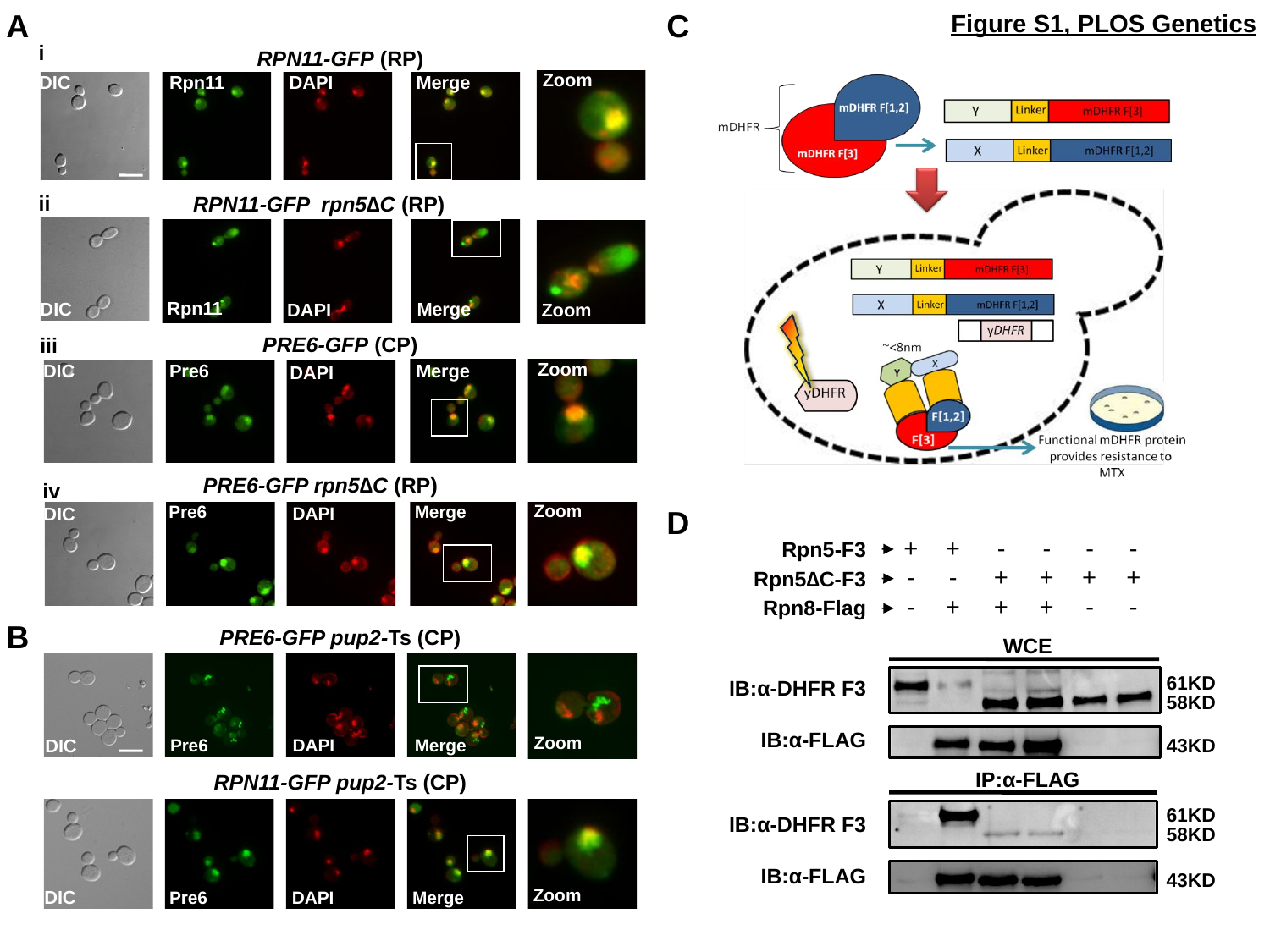

A
C
Figure S1, PLOS Genetics
i
RPN11-GFP (RP)
Zoom
DIC
Rpn11
DAPI
Merge
ii
RPN11-GFP rpn5∆C (RP)
Zoom
Rpn11
DIC
Merge
DAPI
PRE6-GFP (CP)
iii
Zoom
DIC
Pre6
Merge
DAPI
PRE6-GFP rpn5∆C (RP)
iv
Zoom
D
Pre6
Merge
DIC
DAPI
+-
-
+
-
+
-
+
+
-
+
+
-+
-
-+
-
Rpn5-F3
Rpn5∆C-F3
Rpn8-Flag
B
PRE6-GFP pup2-Ts (CP)
WCE
61KD
IB:α-DHFR F3
58KD
IB:α-FLAG
Zoom
43KD
DIC
DAPI
Merge
Pre6
IP:α-FLAG
RPN11-GFP pup2-Ts (CP)
61KD
IB:α-DHFR F3
58KD
IB:α-FLAG
43KD
Zoom
DIC
DAPI
Merge
Pre6
