# Supplementary material for: The Protein Quality Control Machinery Regulates Its Misassembled Proteasome Subunits
Source: PLoS Genet. 2015 Apr 28;11(4):e1005178. doi: 10.1371/journal.pgen.1005178 (PMC4412499; doi:10.1371/journal.pgen.1005178)
Supplement: S2 Fig — Logarithmic cells expressing GFP-Rpn5ΔC (YSB1044) were grown in galactose containing media at the semi-permissive temperature (30°C). The localization of GFP-Rpn5ΔC was scored as nuclear, IPOD only, IPOD and JUNQ (pointed out by white arrows), or cells containing more than 2 puncta, probably representing the Q-bodies. A minimum of 200 cells was counted (n>200); error bars show the standard deviation between two independent experiments. Bars, 5 μm. (B) The wt copy of RPN11 in the rpn5ΔC/RPN5 heterozygous diploid re-localized to the nucleus. Logarithmic rpn5ΔC/RPN5 cells at the semi-permissive temperature (30°C), containing the RP subunit Rpn11 fused to GFP (Rpn11), and Hsp42 fused to TFP (Hsp42) (YSB577X4742) were grown in rich medium. Cells were visualized by DIC, GFP and mCherry. (C) Western blot detects the levels of GFP-rpn5ΔC following a GAL1 promoter shut-off chase experiment. The expression of GAL1-GFP-rpn5ΔC (GFP-rpn5ΔC) was induced in the indicated strains (YSB906, YSB908), by growing the cells at the semi-permissive temperature (30°C) in 2% galactose (Gal) for 2 hrs (t-0). Cells were released into 2% glucose to shut-off the expression of GFP-rpn5ΔC, and samples were collected at timely intervals. Glucose was supplemented with 20mM MG132, or with DMSO (control). Protein extracts were immunoblotted (IB) with α-GFP antibody. Ponceau staining of the blotted protein extracts is shown for loading control. (D) Physical interactions between Hsp42 and the misassembled proteasome subunits Rpn8. wt (YSB219), HSP42-GFP/RPN5 (YSB748), and HSP42-GFP/rpn5ΔC (YSB1191) cells grown at the semi-permissive temperature (30°C) were subjected to immunoprecipitation (IP) with an anti-GFP antibody. Whole cell protein extracts (WCE), and IP samples, were subjected to immunoblotting (IB) with anti-GFP and anti-Rpn8 antibodies. (E,F) Rpn5ΔC cytosolic aggregates formation was not affected by the deletions of HSP26, and HSP104. Representative images of logarithmically growing cells expr [file pgen.1005178.s002.pptx]

## Slide 1
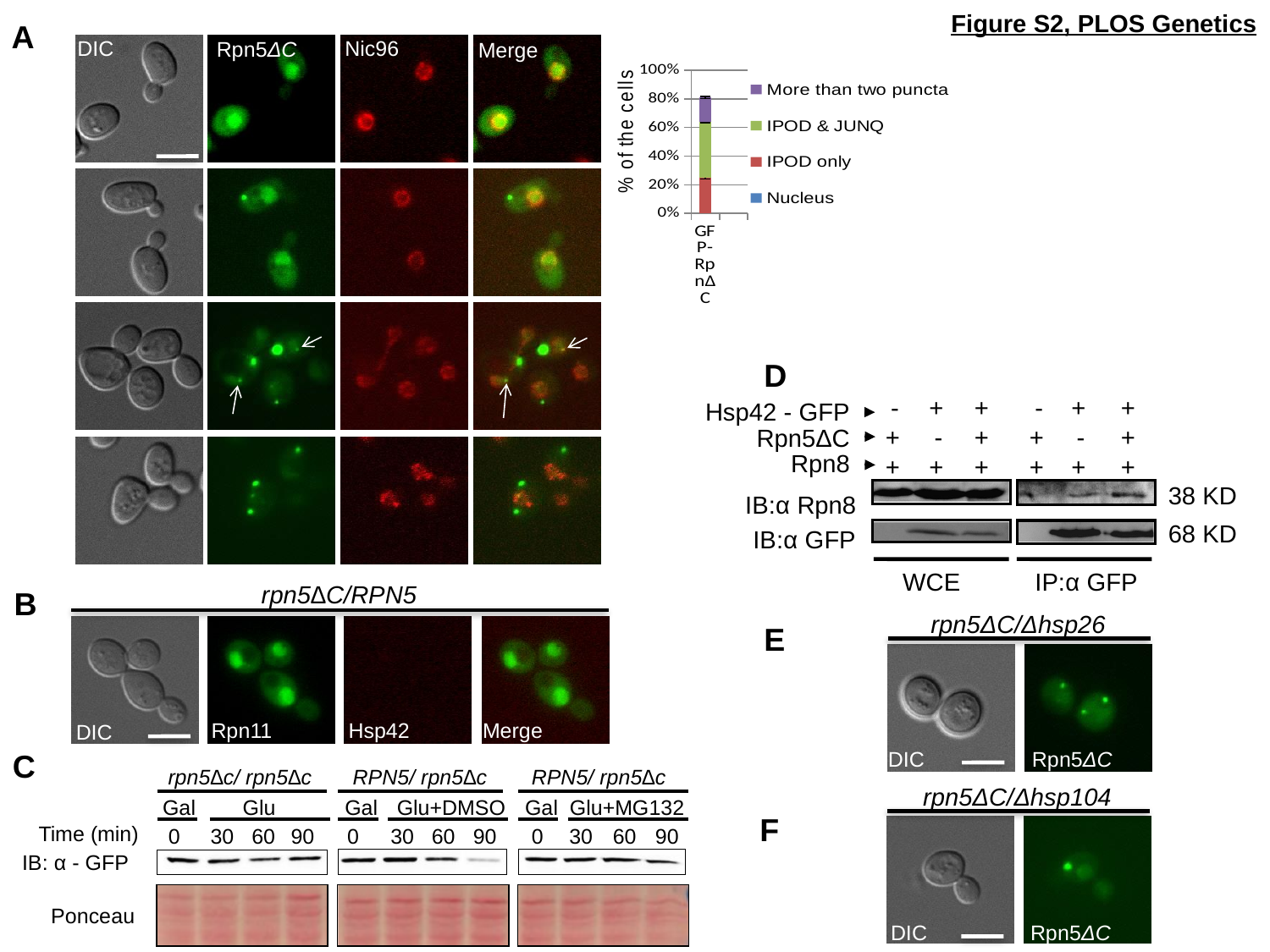

Figure S2, PLOS Genetics
A
### Chart
| Category | Nucleus | IPOD only | IPOD & JUNQ | More than two puncta |
|---|---|---|---|---|
| GFP-Rpn∆C | 0.19 | 0.25 | 0.39 | 0.17 |DIC
Nic96
Rpn5ΔC
Merge
D
++
+
-+
+
+-
+
++
+
-+
+
+-
+
Hsp42 - GFP
Rpn5ΔC
Rpn8
38 KD
IB:α Rpn8
68 KD
IB:α GFP
WCE
IP:α GFP
rpn5∆C/RPN5
Rpn11
Hsp42
Merge
DIC
B
rpn5ΔC/Δhsp26
DIC
Rpn5ΔC
E
C
rpn5∆c/ rpn5∆c
RPN5/ rpn5∆c
RPN5/ rpn5∆c
Gal
Glu
Gal
Glu+DMSO
Gal
Glu+MG132
Time (min)
0
30
60
90
0
30
60
90
0
30
60
90
IB: α - GFP
Ponceau
rpn5ΔC/Δhsp104
DIC
Rpn5ΔC
F
