# Supplementary material for: The Protein Quality Control Machinery Regulates Its Misassembled Proteasome Subunits
Source: PLoS Genet. 2015 Apr 28;11(4):e1005178. doi: 10.1371/journal.pgen.1005178 (PMC4412499; doi:10.1371/journal.pgen.1005178)
Supplement: S1 Table — (DOCX) [file pgen.1005178.s004.docx]

| Table-S1. Relevant genotype of strains used in this study | | | | |
| --- | --- | --- | --- | --- |
| Strain | Genotype | Reference | | |
| BY4741 | *MAT***a** *his3Δ1 leu2Δ0 met15Δ0 ura3Δ0* | | ([Brachmann et al., 1998](#_ENREF_1)) |  |
| BY4742 | *MAT*α *his3Δ1 leu2Δ0 lys2Δ0 ura3Δ0* | | ([Brachmann et al., 1998](#_ENREF_1)) |  |
| YSB 655 | *MAT***a**/α *rpn5Δ45-DHFR(F3)-HYGB/ RPN5* | | This study |  |
| YSB 688 | *MAT*α *rpn5Δ34-DHFR(F3)-HYGB* | | This study |  |
| YSB 226 | *MAT*α *rpn5Δc-DHFR(F3)-HYGB* | | This study |  |
| YSB 741 | *MAT*α *rpn5Δ25-DHFR(F3)-HYGB* | | This study |  |
| YSB 863 | *MAT*α *rpn5Δ20-DHFR(F3)-HYGB* | | This study |  |
| YSB 861 | *MAT*α *rpn5Δ15-DHFR(F3)-HYGB* | | This study |  |
| YSB 758 | *MAT*α *rpn5Δ10-DHFR(F3)-HYGB* | | This study |  |
| YSB 756 | *MAT*α *rpn5Δ5-DHFR(F3)-HYGB* | | This study |  |
| YSB 243 | *MAT*α *RPN5-DHFR(F3)-HYGB* | | This study |  |
| YSB 671 | *MAT***a** *RPN3-DHFR(F1,2)-clonNAT* | | This study |  |
| YSB 244 | *MAT***a** *RPN6-DHFR(F1,2)-clonNAT* | | This study |  |
| YSB 677 | *MAT***a** *RPN7-DHFR(F1,2)-clonNAT* | | This study |  |
| YSB 245 | *MAT***a** *RPN8-DHFR(F1,2)-clonNAT* | | This study |  |
| YSB 670 | *MAT***a** *RPN10-DHFR(F1,2)-clonNAT* | | This study |  |
| YSB 672 | *MAT***a** *RPN11-DHFR(F1,2)-clonNAT* | | This study |  |
| YSB 689 | *MAT***a** *RPN12-DHFR(F1,2)-clonNAT* | | This study |  |
| YSB 1061 | *MAT***a** *rpn5Δ34-DHFR(F3)-HYGB RPN8-*FLAG*-KmX* | | This study |  |
| YSB 59 | *MAT***a**/α *CLN3- DHFR(F3)- HYGB CDC19- DHFR(F1,2)-clonNAT* | | Gift from [Michnick SW](http://www.ncbi.nlm.nih.gov/pubmed/?term=Michnick%20SW%5BAuthor%5D&cauthor=true&cauthor_uid=18467557) laboratory |  |
| YSB 60 | *MAT***a**/α *MCK1-DHFR(F3)- HYGB CDC19- DHFR(F1,2)-clonNAT* | | Gift from [Michnick SW](http://www.ncbi.nlm.nih.gov/pubmed/?term=Michnick%20SW%5BAuthor%5D&cauthor=true&cauthor_uid=18467557) laboratory |  |
| YSB 675 | *MAT*α *RPN5-DHFR(F3)-HYGB RPN8-*FLAG*-KmX* | | This study |  |
| YSB 715 | *MAT***a** RPN5-GFP*-URA3 Δhsp26-clonNAT* | | This study |  |
| SB147 | *MAT***a** *KmX-GAL1-*GFP*-RPN5* | | This study |  |
| SB148 | *MATa KmX-GAL1-*GFP*- rpn5Δc-URA3* | | This study |  |
| YSB 868 | *MAT***a** *KmX-GAL1-*GFP*-rpn5Δc-URA3 Δhsp42-KmX* | | This study |  |
| YSB 1045 | *MAT***a** *KmX-GAL1-*GFP*-rpn5Δc-URA3 Δhsp42-KmX*  *NIC96-*mCherry*-HYGB* | | This study |  |
| YSB 1044 | *MAT***a** *KmX-GAL1-*GFP-*rpn5Δc-URA3 NIC96-*mCherry*-HYGB* | | This study |  |
| YSB 903 | *MAT*α *KmX-GAL1-GFP-rpn5Δc-URA3*  *HSP26-*mCherry*-HYGB* | | This study |  |
| YSB 747 | *MAT***a** *KmX-GAL1-GFP-rpn5Δc-URA3 HSP104-TFP-clonNAT* | | This study |  |
| YSB 792 | *MAT***a** *rpn5Δc-DHFR(F3)-HYGB HSP42-TFP-clonNAT HSP104-GFP-HIS3* | | This study |  |
| SB 162 | *MAT***a** *RPN11-GFP-HIS3 rpn5Δc-URA3 Δpep4* | | This study |  |
| YSB 1002 | *MAT***a** *RPN11-GFP::HIS3 rpn5Δc-URA3 Δhsp42::KMX Δpep4* | | This study |  |
| YSB 1004 | *MAT***a***/α KmX-GAL1-GFP-rpn5Δc-URA3/ RPN5*  *pESC-GAL1-RNQ1-mCherry [LEU2]* | | This study |  |
| YSB 1056 | *MAT***a**/α *RPN5-RFP-KMX/ KmX-GAL1-GFP-rpn5Δc-URA3* | | This study |  |
| YSB 908 | *MAT***a**/α *KmX-GAL1-GFP-rpn5Δc-URA3/ RPN5*  *Δerg6-clonNAT/ Δerg6-clonNAT* | | This study |  |
| YSB 906 | *MAT***a**/α *KmX-GAL1-GFP-rpn5Δc-URA3/ KmX-GAL1-GFP-rpn5Δc-URA3 ade2-1-clonNAT PRE6-mCherry-HYGB* | | This study |  |
| SB 223 | *MAT***a** *RRE6-GFP-HIS3 pup2-Ts-URA3* | | This study |  |
| SB 220 | *MATa RPN11-GFP-HIS3 pup2-Ts-URA3* | | This study |  |
| SB 160 | *MAT***a** *leu2-3,112 trp1-1 can1-100 ura3-1 ade2-1 his3-11,15 bar1-LEU2 pep4-TRP1 PRE6-GFP-HIS3* | | Gift from T. Mayor laboratory |  |
| SB 163 | *MAT***a** *PRE6-GFP-HIS rpn5Δc-URA3* | | This study |  |
| SB 158 | *MAT***a** *leu2-3,112 trp1-1 can1-100 ura3-1 ade2-1 his3-11,15 bar1-LEU2 pep4-TRP1 RPN11-GFP-HIS3* | | Gift from T. Mayor laboratory |  |
| YSB 106 | *MATα rpn5Δc-URA3 pYES-DEST52-psmd12Δc* [*URA3*] | | This study |  |
| LSB 1 | *MATα rpn5Δc-URA3 pYES-DEST52-psmd12* [*URA3*] | | This study |  |
| LSB 2 | *MATα rpn5Δc-URA3 pYES-DEST52-RPN5* [*URA3*] | | This study |  |
| LSB 3 | *MATα rpn5Δc-URA3 PRS316* [*URA3*] | | This study |  |
| YSB1090 | *MATα KmX-GAL1-GFP-rpn5Δc-URA3 RPN11-TFP-clonNAT* | | This study |  |
| YSB 577 | *MAT***a** *RPN11-GFP-HIS3 rpn5Δc-URA3 HSP42-TFP-clonNAT* | | This study |  |
| YSB 1174 | *MAT***a** *KmX-GAL1-*GFP*-rpn5Δc-URA3 Δhsp104-KmX* | | This study |  |
| YSB 748 | *MAT***a** *HSP42-GFP-HIS3* | | This study |  |
| YSB 1191 | *MAT***a** *HSP42-GFP-HIS3 rpn5Δc-DHFR(F3)-HYGB* | | This study |  |
